# The immunosuppressive tumor microenvironment in post-transplant lymphoproliferative disorder: pathogenesis and novel therapeutic frontiers

**DOI:** 10.3389/fimmu.2026.1774164

**Published:** 2026-04-07

**Authors:** Andrea Dello Strologo, Chiara De Liso, Giulia Scarsella, Alessia Trotta, Claudia Strazza, Letizia Donatone, Francesco Pesce, Giuseppe Grandaliano

**Affiliations:** 1 Department of Nephrology and Dialysis, Azienda USL Roma 6, Albano Laziale, Italy; 2 Department of Translational Medicine and Surgery, Università Cattolica del Sacro Cuore, Rome, Italy; 3 Division of Renal Medicine, “Hospital Isola Tiberina-Gemelli Isola”, Rome, Italy; 4 Nephrology, Dialysis and Transplantation Unit, Fondazione Policlinico Universitario A.Gemelli IRCCS, Rome, Italy

**Keywords:** Epstein-Barr virus (EBV), immunotherapy, kidney transplantation, post-transplant lymphoproliferative disorder (PTLD), tumor microenvironment

## Abstract

Post-transplant lymphoproliferative disorder (PTLD) in kidney transplantation is increasingly recognized not merely as a passive consequence of systemic immunosuppression, but as a malignancy driven by an actively engineered, immunosuppressive tumor microenvironment (TME). This review explores the sophisticated mechanisms by which Epstein-Barr Virus (EBV) remodels the local cellular landscape, detailing how the viral oncoprotein LMP1 acts as a master regulator to upregulate immune checkpoints like PD-L1 and orchestrate the recruitment of M2-polarized macrophages and regulatory T cells. We further highlight the emerging role of extracellular vesicles (exosomes) as vesicles for viral microRNAs, enabling the tumor to condition immune cells at a distance and establish a tolerogenic niche. These viral strategies are contrasted with the distinct pathogenesis of late-onset, EBV-negative PTLD, which relies on genomic instability rather than viral immunomodulation. Finally, we evaluate how this deepened understanding of the TME is transforming therapeutic paradigms, moving from standard reduction of immunosuppression toward targeted interventions, such as EBV-specific adoptive T-cell therapies (Tabelecleucel) and CAR-T cells, designed to dismantle the tumor’s protective architecture while minimizing the high risk of allograft rejection associated with checkpoint inhibitors.

## 1 Introduction

Organ transplantation represents the gold standard therapeutic strategy for most end-stage organ failure conditions, such as those involving the heart, lungs, liver and kidneys. Specifically, kidney transplantation (KT) guarantees the best possible treatment for patients with end-stage kidney disease in terms of both survival and quality of life compared with patients undergoing dialysis. However, solid-organ transplant recipients necessitate antirejection immunosuppressive therapies to reduce rejection risk and improve graft survival. Such therapies predispose patients to developing malignancies induced both by immunosuppression and by viral infections with oncogenic potential (1, 2).

Transplant patients have an increased risk of developing all types of malignancies. The post-transplant lymphoproliferative disorders (PTLD) comprise a heterogeneous group of conditions characterized by uncontrolled proliferation of lymphoid cells occurring in the post-transplant period: recipient Epstein–Barr virus (EBV) seronegative and the intensity of immunosuppression are among the main risk factors (2). PTLD is increasingly recognized not merely as a passive consequence of systemic immunosuppression, but as a malignancy driven by an actively engineered, immunosuppressive tumor microenvironment (TME). Overall, organ transplantation creates a tumor-permissive microenvironment by combining iatrogenic immunosuppression with EBV-driven immune evasion, elevated immunosuppressive cytokines, and impaired dendritic- and T-cell function. These alterations collectively promote the survival and uncontrolled proliferation of EBV-infected B cells, facilitating PTLD development (3).

Therefore, it is critical to gain a deeper understanding of the underlying mechanisms that drive PTLD development and the resulting perturbations in biological processes. In this review, we summarize and discuss the existing literature to clarify the incidence and management of PTLD in KT; furthermore, we highlight the critical role of the tumor/immunologic microenvironment in the pathogenesis of PTLD and future perspectives regarding new therapeutic strategies.

## 2 Epidemiology and pathogenesis

### 2.1 Epidemiology and risk factors

The incidence of PTLD is up to 20% among solid-organ transplant recipients, and after skin cancers, EBV-positive PTLDs, as previously mentioned, are the most frequent malignancies in transplant recipients (2). The onset varies according to the transplanted organ, this difference may be related to the amount of lymphoid tissue transplanted and to the intensity of immunosuppression required to prevent acute graft rejection, which depends on the specific organ (2, 4). Specifically, multiorgan and intestinal transplantations show the highest incidence, reaching up to 20% within 10 years post-transplant. Other examples include heart and lung transplants, which stand at approximately 4%, and liver transplants at around 3%, also at the 10-year mark. In contrast, kidney transplantation accounts for the lowest incidence, typically ranging from 0.8% to 2.5% over 10 years, with standardized incidence ratios of approximately 6–12 compared to the general population (2, 4).

According to the Organ Procurement and Transplant Network database in the USA, the incidence density of PTLD after kidney transplantation has been reported as 1.58 per 1000 person-years, while in the United Kingdom the incidence density for the non-Hodgkin lymphoma PTLD subtype is 2.6 per 1000 person-years (1). The ANZDATA registry reported a 25-year cumulative incidence of PTLD of 3.3% in adults and 3.6% in children, adjusted for competing risks such as death (5).

Several factors increase the risk of PTLD, and many act synergistically, potentially raising the risk up to 300-fold compared with recipients without risk factors. EBV-seronegative status at the time of transplantation increases the subsequent risk of PTLD more than 12-fold relative to EBV-positive recipients; the highest risk is observed in those who acquire primary EBV infection after transplantation (likely due to receiving an organ from an EBV-positive donor) (4).

Furthermore, EBV status is strongly associated with the temporal pattern of PTLD onset. EBV-positive PTLD typically emerges early, often within the first months after transplantation, reflecting primary EBV infection or uncontrolled reactivation in the context of intense immunosuppression. In contrast, EBV-negative PTLD generally develops late, frequently years after transplantation, and is thought to arise through mechanisms independent of direct viral oncogenesis, including accumulated genomic instability and chronic antigenic stimulation (6).

In transplant recipients, high-intensity immunosuppressive regimens represent a significant risk factor for the development of PTLD. In particular, T-cell depletion with ATG, high doses of calcineurin inhibitors (CNI), and the use of mycophenolate mofetil (MMF) are associated with an increased risk, with studies reporting up to 62.5% central nervous system involvement in patients treated with MMF (7). These drugs reduce immune surveillance against EBV, facilitating the proliferation of infected B cells. Indeed, the reduction of immunosuppression, used as the first line of treatment, is effective in only 23–50% of cases, with better outcomes in early-onset disease (7). Conversely, antiproliferative agents such as mammalian Target of Rapamycin (mTOR) inhibitors (e.g., everolimus) have demonstrated remission rates of up to 79% when introduced after CNI reduction, representing a potential protective factor and helping to preserve graft function (8).

### 2.2 Pathogenesis and classification

Several subtypes of PTLD exist and have been classified by the WHO Histological Classification, which defines four key categories (2, 9). Early lesions (including plasmacytic hyperplasia and infectious mononucleosis–like PTLD) represent the least aggressive category. They are almost always EBV-driven, non-destructive, and may regress spontaneously. The Polymorphic PTLD is characterized by a destructive but mixed lymphoid infiltrate that does not yet meet the full criteria for malignancy. Monomorphic PTLD (mPTLD) is the most common and aggressive subtype, as it fulfills the criteria for conventional non-Hodgkin lymphomas. The large majority of mPTLDs are of B-cell origin, predominantly presenting as Diffuse Large B-cell Lymphoma. These forms carry the worst prognosis, especially in kidney transplant recipients. A final, rare category is the Classic Hodgkin Lymphoma-like PTLD (4, 10).

The underlying pathogenesis in the majority of cases is driven by the EBV. The suppression of T-cell immunity to prevent allograft rejection eliminates the host’s immunosurveillance, allowing for the uncontrolled expansion of EBV-infected B cells. EBV-driven transformation relies on the sequential expression of viral antigens (latency programs) within the B cell. These programs are fundamental to viral persistence within host B cells, immune evasion, and oncogenesis, distinguishing themselves by the specific viral proteins expressed, which confer different levels of proliferation and immunogenicity (11). The most highly proliferative is Latency Type III (or the Growth Program), commonly found in early and polymorphic PTLD, where the virus expresses all six Nuclear Antigens (EBNA1-6) and all Latent Membrane Proteins (LMP1, LMP2A/B). This robust expression provides the strongest proliferative signal, using the oncogene LMP1 to mimic constitutive CD40 signaling and EBNA2 as a master transactivator for viral and cellular genes. The Latency Type II in contrast, maintains only EBNA1, LMP1, and LMP2A. This profile, observed in some monomorphic PTLD cases, allows the B cell to survive and differentiate while significantly reducing the immunogenic load due to the silencing of highly recognizable proteins like EBNA2-6. The most restricted program, Latency Type I, expresses primarily only EBNA1 (essential for genome replication) and non-coding Epstein-Barr Virus-Encoded RNAs (EBERs). This minimal expression is characteristic of resting memory B cells and highly malignant lymphomas like Burkitt lymphoma. The selective pressure exerted by therapeutic immunosuppression and host immune response often drives the clonal evolution of PTLD from the highly immunogenic Type III toward the less immunogenic Type II or I phenotypes, reflecting the biological progression toward a more aggressive, immune-evasive state (4, 6, 11).

**Figure 1:**
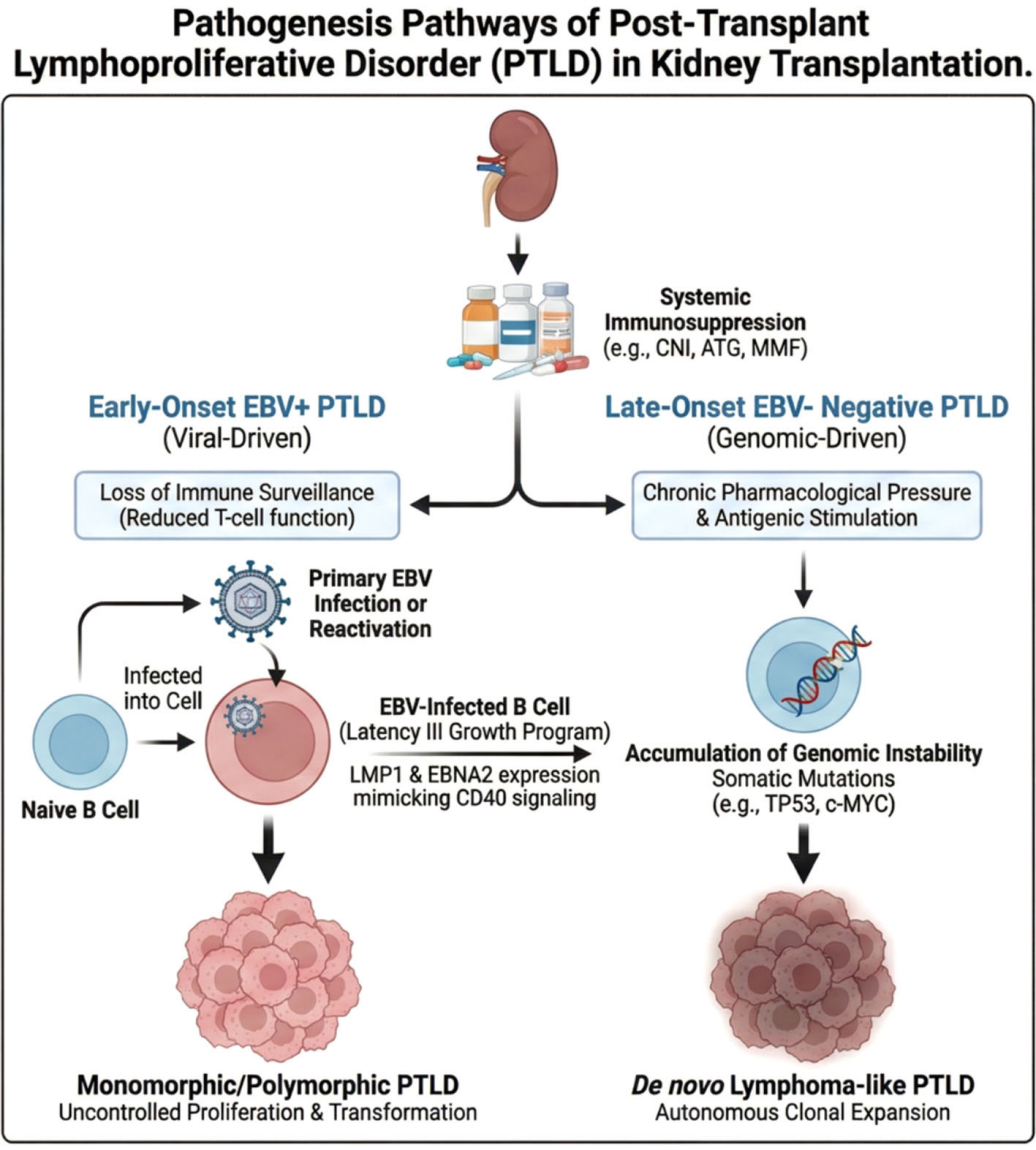
Pathogenesis pathways of post-transplant lymphoproliferative disorder (PTLD) in kidney transplantation. The diagram illustrates the divergent mechanisms driving PTLD under systemic immunosuppression. The viral-driven pathway (left) characterizes early-onset EBV-positive PTLD, where loss of T-cell surveillance facilitates EBV reactivation, expression of viral oncogenes (LMP1 and EBNA2), and subsequent uncontrolled B-cell proliferation. Conversely, the genomic- driven pathway (right) characterizes late-onset EBV-negative PTLD, driven by chronic pharmacological pressure and antigenic stimulation. This environment fosters genomic instability and somatic mutations (e.g., TP53, c-MYC), leading to autonomous clonal expansion indistinguishable from de novo lymphomas. Abbreviations List: ATG, Anti-thymocyte globulin; CD40, Cluster of differentiation 40; CNI, Calcineurin inhibitors; c-MYC, Cellular MYC; EBNA2, Epstein-Barr nuclear antigen 2; EBV, Epstein- Barr virus; LMP1, Latent membrane protein 1; MMF, Mycophenolate mofetil; PTLD, Post-transplant lymphoproliferative disorder; TP53, Tumor protein p53.

In some cases, PTLD develops in EBV-negative patients, typically presenting in long course kidney transplant. These forms are not virally driven and, in contrast, are governed by alternative oncogenic mechanisms resulting from prolonged immunosuppression. The pathogenesis here is characterized by the accumulation of genomic alterations and significant chromosomal instability. These genetic events include somatic mutations in key tumor suppressors and oncogenes such as *TP53* and *c-MYC*. Consequently, EBV-negative PTLD exhibits molecular and clinical characteristics highly similar to *de novo* lymphomas arising in the general immunocompetent population (4, 11) (**Figure 1**).

## 3 The immunosuppressive tumor microenvironment in PTLD

The development of PTLD is not merely a consequence of systemic suppression, but critically depends on the ability of EBV-infected B cells to actively shape a protective, immunosuppressive TME locally. This TME is characterized by a concerted failure of both adaptive and innate immunity, facilitating uncontrolled proliferation (12).

### 3.1 The immunosuppressive microenvironment of PTLD EBV-driven

The establishment of a suppressive cellular infiltrate in PTLD after kidney transplantation is predominantly orchestrated by myeloid-derived suppressor cells (MDSCs), particularly the monocytic subset (M-MDSCs), which accumulate in response to chronic immunosuppression and EBV-driven inflammation. These represent a heterogeneous population of immature myeloid-lineage cells characterized by potent immunosuppressive activity. Within the PTLD tumor microenvironment, M-MDSCs expand significantly in kidney transplant recipients and exert powerful effects through multiple complementary pathways. This pathological remodeling of the TME is marked by a significant shift in cellular abundance, where a profound lymphopenia of effector Natural killer (NK) and T cells is accompanied by a massive, progressive infiltration of suppressive myeloid populations (13, 14). They suppress anti-tumor immunity by depleting L-arginine via arginase-1 production, which is essential for T-cell receptor expression and proliferation, thereby impairing effector T-cell function (15, 16). Additionally, M-MDSCs generate reactive oxygen species and nitric oxide through inducible nitric oxide synthase, creating a metabolically hostile microenvironment that directly inhibits T-cell activation and cytotoxic capacity (15, 17, 18). The expression of indoleamine 2,3-dioxygenase further contributes to this immunosuppressive state by catabolizing tryptophan to kynurenine, which suppresses T-cell proliferation and promotes regulatory T-cell (Treg) activation (19).

A critical feature of the PTLD microenvironment is the bidirectional crosstalk between M-MDSCs and Tregs, which serves to amplify local immunosuppression. M-MDSCs isolated from kidney transplant recipients demonstrate high efficiency in expanding CD4+CD25+Foxp3 Tregs *in vitro*, and their accumulation over time post-transplantation correlates linearly with increased Treg frequencies *in vivo* (14). This expansion is mediated by several mechanisms, including the secretion of transforming growth factor-β (TGF-β) and IL-10, both of which are upregulated in the PTLD microenvironment and promote Treg differentiation (16, 20). Furthermore, tumor-infiltrating M-MDSCs secrete CCR5 ligands (CCL3, CCL4, and CCL5) to selectively recruit CCR5-expressing Tregs to the tumor site, creating a concentrated immunosuppressive niche (21). This chemokine-mediated recruitment is particularly relevant in PTLD, where the spatial proximity between M-MDSCs and Tregs facilitates sustained immune evasion and enables the persistence of EBV-infected B cells (21, 22).

Phenotypically, M-MDSCs in PTLD are identified by the CD11b+CD33+HLA-DR-lowCD14 profile (13, 14). These cells show reduced antigen-presenting capacity due to the downregulation of HLA-DR and costimulatory molecules, further impairing their ability to activate effector T cells (19). Paradoxically, M-MDSCs express programmed death-ligand 1 (PD-L1), which engages the programmed death protein 1 (PD-1) receptor on T cells to inhibit activation and promote T-cell exhaustion (23, 24). This PD-L1 expression is notably elevated in EBV-positive PTLD cases, correlating with increased immunosuppression and disease progression (25). EBV infection profoundly influences this polarization; EBV-induced cytokines like IL-6 and IL-10 drive M-MDSC activation, while the EBV latent membrane protein 1 (LMP-1) directly regulates the expression of immunosuppressive markers such as PD-L1, CD163, and CD206 to promote an M2-like phenotype (25–28).

**Figure 2:**
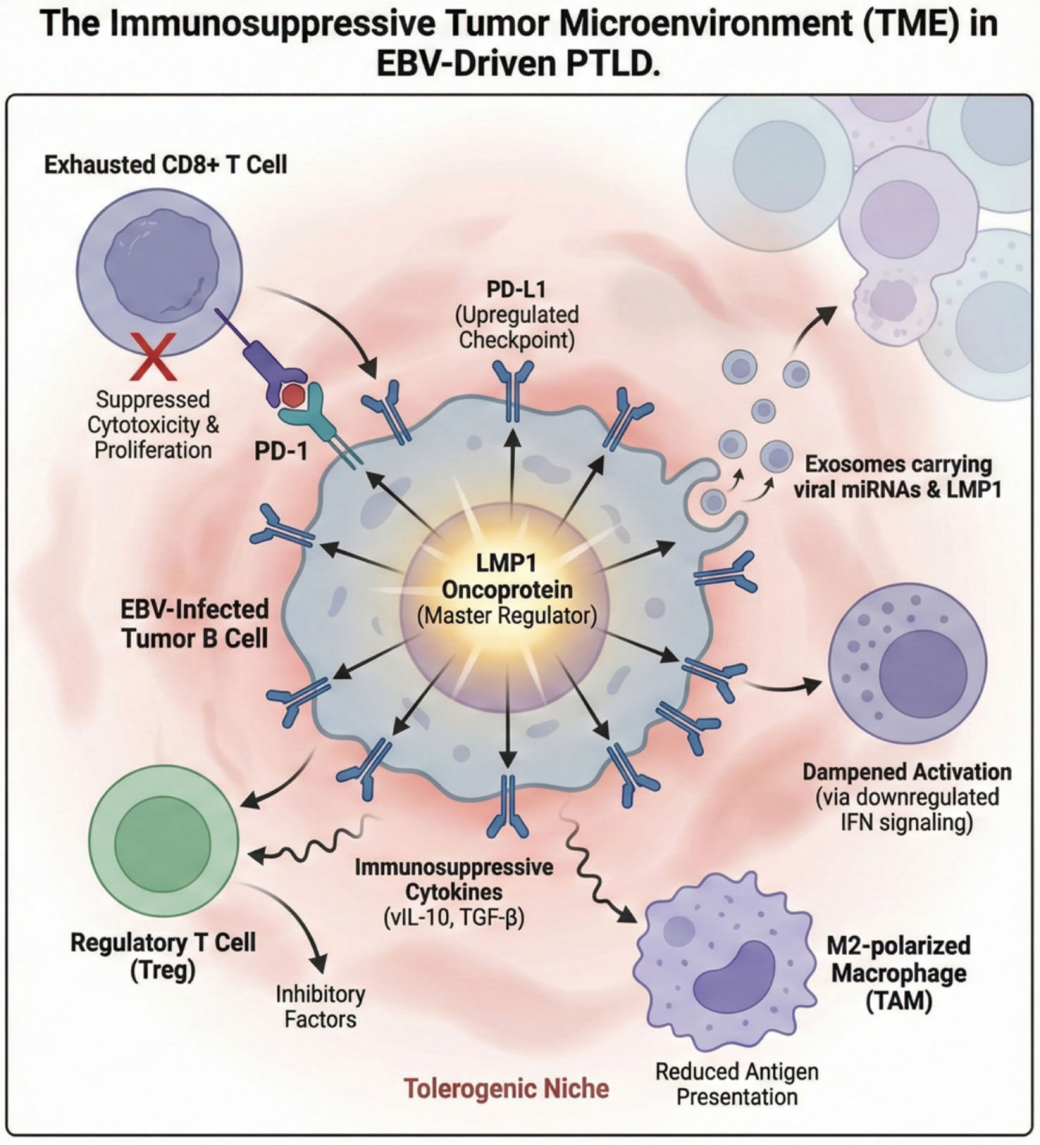
The immunosuppressive tumor microenvironment (TME) in EBV-driven post-transplant lymphoproliferative disorder (PTLD). This diagram illustrates the mechanisms by which the EBV-infected tumor B cell actively engineers a tolerogenic niche, with the LMP1 oncoprotein acting as a master regulator. The tumor cell upregulates the immune checkpoint ligand PD-L1, engaging PD-1 on CD8+ T cells to induce exhaustion and suppress cytotoxicity. Concurrently, the secretion of immunosuppressive cytokines, such as vIL-10 and TGF-β, facilitates the recruitment of regulatory T cells (Tregs) and promotes the polarization of macrophages into an immunosuppressive M2 phenotype (TAMs). Additionally, tumor-derived exosomes transporting viral miRNAs and LMP1 dampen immune activation in the surrounding microenvironment by downregulating interferon (IFN) signaling pathways. CD8+, Cluster of differentiation 8 positive; EBV, Epstein-Barr virus; IFN, Interferon; LMP1, Latent membrane protein 1; miRNAs, microRNAs; PD-1, Programmed cell death protein 1; PD-L1, Programmed death-ligand 1; PTLD, Post-transplant lymphoproliferative disorder; TAM, Tumor-associated macrophage; TGF-β, Transforming growth factor-beta; TME, Tumor microenvironment; Treg, Regulatory T cell; vIL-10, viral interleukin-10.

In addition to MDSCs, tumor-associated macrophages contribute significantly to the suppressive infiltrate, frequently exhibiting an M2-like phenotype (CD163+, CD206+, c-Maf+) and secreting IL-10 and TGF-β (26, 29). However, the macrophage compartment remains heterogeneous, with M1 polarization suggesting an active but ineffective immune response in EBV-positive cases (26). NK cells also exhibit profound dysfunction and exhaustion in EBV-positive PTLD (6, 30, 31). Patients often present with profound NK-cell lymphopenia and altered subsets, such as the accumulation of CD56-CD16 cells at the expense of functionally competent subsets (31). EBV employs sophisticated mechanisms to evade NK cells, including LMP-1 peptide variants presented by HLA-E that elicit inhibitory NKG2A+ responses (32–34) and the downregulation of NKG2D ligands through EBNA1 expression (35). Although entry into the lytic cycle can sensitize cells to NK killing via MICB upregulation (25), resistance is often acquired in the late lytic cycle through the viral Bcl-2 homologue BHRF1 (36). (**Figure 2**).

**Figure 3:**
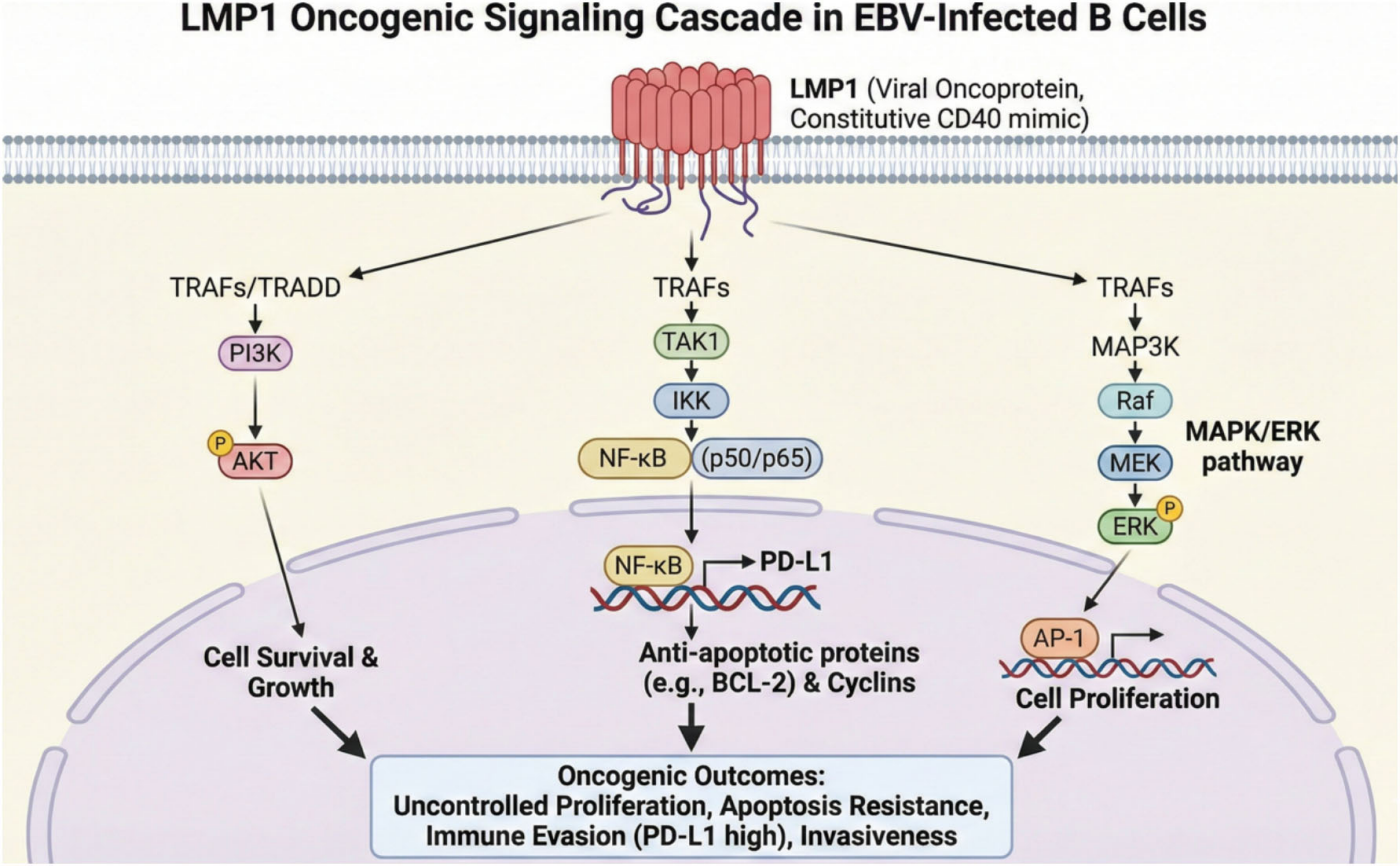
LMP1 oncogenic signaling cascade in EBV-infected B cells. This diagram illustrates the constitutive activation of downstream signaling pathways by the viral oncoprotein LMP1, which mimics CD40 signaling. LMP1 recruits tumor necrosis factor receptor-associated factors (TRAFs) and TRADD to activate three key pathways: the PI3K/AKT pathway, leading to cell survival and growth; the NF-κB pathway (via TAK1 and IKK), which upregulates anti-apoptotic proteins (e.g., BCL-2), cyclins, and the immune checkpoint ligand PD-L1; and the MAPK/ERK pathway (via MAP3K, Raf, and MEK), which activates the AP-1 transcription factor to promote cell proliferation. Collectively, these pathways drive oncogenic outcomes, including uncontrolled proliferation, resistance to apoptosis, immune evasion, and invasiveness. AKT, Protein Kinase B; AP-1, Activator protein 1; BCL-2, B-cell lymphoma 2; CD40, Cluster of differentiation 40; EBV, Epstein-Barr virus; ERK, Extracellular signal-regulated kinase; IKK, IκB kinase; LMP1, Latent membrane protein 1; MAP3K, Mitogen-activated protein 3 kinase; MAPK, Mitogen-activated protein kinase; MEK, Mitogen-activated protein kinase; NF-κB, Nuclear factor kappa-light-chain-enhancer of activated B cells; P, Phosphate group; PD-L1, Programmed death-ligand 1; PI3K, Phosphoinositide 3-kinase; Raf, Rapidly Accelerated Fibrosarcoma; TAK1, Transforming growth factor-β-activated kinase 1; TRADD, tumor necrosis factor receptor-associated factors; TRAFs, Tumor necrosis factor receptor-associated factors.

The core of EBV-positive PTLD is the uncontrolled proliferation of B lymphocytes, a process driven by specific EBV latency programs, most notably Latency III. This program involves the expression of the full spectrum of latent viral genes, including EBNA1, EBNA2, EBNA3A/B/C, and LMP1/2A, which collectively hijack normal B-cell developmental pathways to promote transformation (2, 6, 37). While Latency III B cells theoretically present a vast array of immunogenic viral antigens (including EBNAs and LMPs), the efficacy of this antigen presentation is completely undermined by the suppressive microenvironment they actively construct (6, 38). Central to this process is LMP1, which acts as a constitutively active viral analogue of CD40 (2, 39). By mimicking CD40 signaling, LMP1 activates critical growth and survival pathways, particularly Nuclear Factor kappa-light-chain-enhancer of activated B cells (NF-κB), through the direct recruitment of signaling adaptors such as TRAF6 and other TRAF molecules (39–41). The signaling capacity of LMP1 is localized within its C-terminal activation regions, CTAR1 and CTAR2; while both are important for B-cell transformation, CTAR2-mediated signaling is strictly required for the survival of EBV-immortalized cells (42). Through these domains, LMP1 triggers multiple downstream cascades, including the JNK/AP-1 and PI3K/Akt pathways, which induce anti-apoptotic proteins such as BCL-2 and Bcl-xL. Furthermore, LMP1 promotes cell cycle progression by enhancing cyclin-dependent kinase 2 activity and the phosphorylation of retinoblastoma protein, while simultaneously inhibiting the cell cycle suppressors p16 and p27 (40, 43)(**Figure 3**).

Beyond driving intrinsic cell proliferation, LMP1 plays a pivotal role in modulating the tumor microenvironment to favor immune evasion. It directly upregulates PD-L1 expression on EBV-infected B cells, with LMP1-positive cells displaying significantly higher levels of this checkpoint ligand than their LMP1-negative counterparts within the same tumor, thereby inhibiting T-cell activation (44). LMP1 also orchestrates the production of immunosuppressive cytokines, most notably IL-10, via the activation of both p38 MAPK and PI3K pathways. In this context, IL-10 serves a dual purpose: it acts as an autocrine growth factor for EBV-transformed B cells and contributes to a suppressive milieu (44, 45). Recent studies suggest that LMP1 acts as more than a proliferative trigger, actively remodeling B-cell oncometabolism to prioritize *de novo* purine biosynthesis. By driving the accumulation of xanthosine-5-phosphate (XMP) via the IMPDH pathway, the virus establishes a metabolic dependency that renders EBV-transformed cells uniquely sensitive to the disruption of this axis (46). Additionally, LMP1 promotes the expansion of both conventional CD25+ and unconventional CD25- regulatory T cells that express latency-associated peptide and PD-1 in a PD-L1-dependent manner. The reach of LMP1 extends even beyond the infected cell itself, as it can be secreted within exosomes to directly suppress T-cell proliferation and NK-cell cytotoxicity through specific immunosuppressive peptide sequences in its transmembrane domain (44).

Current evidence confirms that EBV-infected B cells also utilize Extracellular Vesicles (EVs), particularly exosomes, as a sophisticated, non-contact dependent mechanism of immune evasion. These small vesicles function as shuttles, transporting viral components such as LMP1 and viral microRNAs (miRNAs) to distant immune and stromal cells within the TME (38, 47–49). Exosomal LMP1 and miRNAs are capable of altering the function of recipient cells, promoting proliferation, migration, differentiation, and transcriptional remodeling to support an immunosuppressive phenotype (38). Specifically, exosomal LMP1 activates ERK, AKT, and NF-κB pathways in recipient cells, favoring growth and invasiveness, while exosomal viral miRNAs suppress host genes involved in both innate and adaptive immune responses, reducing effector T lymphocyte diversity and infiltration (38). These exosome-mediated mechanisms represent a key strategy for EBV to evade immune control and promote the pathogenesis of malignancies like PTLD (50) (**Figure 3**).

The survival and malignant transformation of B cells in PTLD are ultimately the result of synergistic effects among multiple viral oncoproteins. LMP1, in conjunction with EBNA2, constitutes the minimal set of EBV genes sufficient for the transformation of human primary B cells (51). This oncogenic synergy is complemented by LMP2A, which provides surrogate B-cell receptor signals, activates the Ras/PI3K/Akt pathway, and induces Bcl-xL to promote survival while blocking lytic cycle reactivation (43, 52). Furthermore, EBNA3A promotes transformation by phenocopying early B-cell factor 1 activities and inhibiting plasma cell differentiation (53). Together with EBV-encoded microRNAs that provide additional anti-apoptotic and proliferative signals, these viral proteins establish a self-sustaining program independent of normal cellular activation signals (43, 54). The combined action of M-MDSCs, TAMs, dysfunctional NK cells, and Tregs, orchestrated by these EBV-encoded oncoproteins, establishes a tumor microenvironment that is highly resistant to immune surveillance, creating a permissive niche for EBV-driven lymphomagenesis in kidney transplant recipients (6, 15, 17, 20, 30)(**Table 1**).

**Table 1:** Key cellular and molecular components of the tumor microenvironment (TME) in Epstein–Barr virus (EBV)-positive post-transplant lymphoproliferative disorder (PTLD) after kidney transplantation and their main functional roles, markers, and potential therapeutic implications.

TME component	Main role in EBV+ PTLD	Key markers / mediators	Potential therapeutic implications
EBV-infected PTLD B cells	Central driver of disease; LMP1/LMP2 and other latent proteins mimic chronic CD40/BCR signaling, sustaining proliferation and survival and orchestrating an immunosuppressive niche.	EBV latency II–III programs; LMP1, LMP2A/B, EBNA2; MYC, NF-κB, JAK–STAT activation.	Direct debulking (rituximab ± chemotherapy); modulation of immunosuppression (RIS, switch to mTORi); EBV-specific T-cell therapies (e.g. tabelecleucel); anti-CD19/CD20 CAR-T in selected cases.
CD8^+^ cytotoxic T cells	Functionally exhausted by chronic antigen exposure and systemic immunosuppression; reduced cytotoxic clearance of EBV+ B cells and impaired antiviral control.	PD-1, TIM-3, LAG-3 upregulation; decreased granzyme B / perforin; exhausted transcriptional profile.	Careful reduction of global immunosuppression to partially restore T-cell function; adoptive EBV-specific CTLs; experimental combinations with checkpoint blockade in highly selected, life-threatening cases (with high rejection risk).
CD4^+^ T cells and Tregs	Skewing toward regulatory/Th2-like profiles promotes tolerance to the tumor, dampening effective anti-lymphoma immunity.	FOXP3^+^ Tregs; high IL-10, TGF-β; CTLA-4, ICOS; Th2 cytokines.	Indirectly targeted by tapering calcineurin inhibitors and using mTOR inhibitors (less pro-oncogenic, relatively sparing of anti-tumor immunity); Treg/CTLA-4 modulation largely experimental in SOT due to rejection risk.
NK cells	Numerical and functional impairment limits innate control of EBV-infected B cells and favors PTLD outgrowth.	Decreased NK cell counts; reduced CD16/CD56 cytotoxic subsets; downregulated activating receptors (NKG2D).	Optimizing overall immunosuppression and viral prophylaxis; experimental strategies to enhance NK function or combine NK-based immunotherapies with EBV-targeted approaches.
Tumor-associated macrophages (TAMs, M2-like)	Promote an immunotolerogenic, pro-survival niche via immunosuppressive cytokines, angiogenesis, and matrix remodeling; enriched in EBV+ PTLD and associated with adverse outcome.	CD163^+^, CD68^+^ M2-like TAMs; IL-10, TGF-β, VEGF, Arginase-1; IDO1 expression; mannose receptor.	Prognostic biomarker (high CD163^+^ density); potential target for macrophage-repolarizing or CSF1R-directed therapies; rationale for combining TME-modifying agents with rituximab-based regimens.
Myeloid-derived suppressor cells (MDSC) and other myeloid cells	Suppress T-cell function, consume nutrients (arginine, tryptophan), and generate reactive species that blunt anti-tumor immunity.	CD11b^+^, CD33^+^; Arginase-1, IDO1, ROS, nitric oxide.	Potential targets for experimental MDSC-directed therapies (e.g. metabolic inhibitors, differentiation agents); support the concept of multi-target TME modulation rather than B-cell–only strategies.
Dendritic cells and antigen-presenting cells	Qualitatively altered antigen presentation; may favor tolerance rather than effective priming of EBV-specific T cells.	Reduced co-stimulatory molecules (CD80/CD86); increased PD-L1, IL-10; tolerogenic DC signatures.	Highlight need to avoid “over-suppression” of co-stimulation (e.g. excessive belatacept exposure) in high-risk EBV+ patients; potential target for DC-based vaccines or agonists in experimental settings.
Cytokine and chemokine milieu	Drives cell recruitment, survival and polarization of immune and stromal populations; EBV and LMP1 reshape this network towards chronic inflammation and immune escape.	IL-6, IL-10, TGF-β, BAFF; CXCL13, CCL17, CCL22; NF-κB–driven transcriptional programs.	Candidate serum/plasma biomarkers for disease activity and prognosis; rationale for targeting specific axes (e.g. IL-6/STAT3, BAFF) in future trials.
Immune checkpoints and metabolic brakes	Upregulation of PD-1/PD-L1, IDO1 and other inhibitory pathways consolidates local immune paralysis despite an inflammatory TME.	PD-L1 on tumor and myeloid cells; PD-1 on T/NK cells; IDO1, TIGIT, LAG-3, CTLA-4.	Justify interest in checkpoint inhibitors for refractory PTLD, but their use in SOT is limited by high risk of acute allograft rejection; checkpoint expression may guide highly selected “last-resort” off-label use or future controlled studies.
Stromal cells, vasculature and extracellular matrix	Provide structural and trophic support, favoring lymphoma cell trafficking and survival; help shape hypoxic, nutrient-depleted niches that further suppress immunity.	Cancer-associated fibroblast markers; VEGF-driven neovascularization; hypoxia/HIF-1α signatures; altered collagen and ECM proteins.	Potential role of anti-angiogenic or stroma-targeting agents (still largely theoretical in PTLD); emphasize that PTLD biology is not purely “cell-autonomous” but niche-dependent.
Exosomes and extracellular vesicles from EBV+ cells	Vesicle for LMP1, EBV-encoded miRNAs and immunomodulatory cargos that reprogram immune and stromal cells at distance, amplifying immune escape beyond the tumor bulk.	LMP1^+^ exosomes; EBV-miR-BART family; PD-L1-carrying vesicles; circulating EV signatures.	Emerging biomarkers for early detection, risk stratification and minimal residual disease monitoring; attractive but still experimental targets (e.g. blocking EV release/uptake or miRNA-based therapies).

TME, tumor microenvironment; EBV, Epstein–Barr virus; PTLD, post-transplant lymphoproliferative disorder; KT, kidney transplantation; BCR, B-cell receptor; LMP, latent membrane protein; CAR-T, chimeric antigen receptor T cell; RIS, reduction of immunosuppression; mTORi, mammalian target of rapamycin inhibitor; CTL, cytotoxic T lymphocyte; PD-1, programmed cell death protein 1; TIM-3, T-cell immunoglobulin and mucin-domain containing-3; LAG-3, lymphocyte activation gene-3; FOXP3, forkhead box P3; Treg, regulatory T cell; CTLA-4, cytotoxic T-lymphocyte–associated antigen-4; ICOS, inducible T-cell co-stimulator; Th2, T helper 2; CNI, calcineurin inhibitor; NK, natural killer; NKG2D, natural killer group 2D; TAM, tumor-associated macrophage; VEGF, vascular endothelial growth factor; IDO1, indoleamine 2,3-dioxygenase 1; MDSC, myeloid-derived suppressor cell; ROS, reactive oxygen species; DC, dendritic cell; PD-L1, programmed death-ligand 1; BAFF, B-cell activating factor; NF-κB, nuclear factor kappa-B; STAT3, signal transducer and activator of transcription 3; TIGIT, T cell immunoreceptor with Ig and ITIM domains; ECM, extracellular matrix; HIF-1α, hypoxia-inducible factor 1-alpha; EV, extracellular vesicle; SOT, solid organ transplantation.

### 3.2 Distinct immunological and genomic landscape of EBV-negative PTLD

While the microenvironment of EBV-positive PTLD is actively sculpted by viral proteins, the immunological landscape of EBV-negative forms is fundamentally distinct, reflecting a pathogenesis more akin to *de novo* lymphomas arising in immunocompetent individuals (2, 26, 55). These cases typically manifest significantly later after transplantation, with a median latency of 71–96 months compared to only 4 months for early-onset EBV-positive disease. Rather than being driven by the coordinated expression of viral latency antigens or immunomodulatory proteins, immune evasion in EBV-negative PTLD is sustained by the progressive accumulation of complex genomic instability under the pressure of chronic pharmacological immunosuppression (2, 30, 56).

Genetically, EBV-negative PTLD displays significantly more complex copy-number aberrations than EBV-positive cases. Recurrent alterations include gains of 3/3q and 18q, as well as losses of 6q23/TNFAIP3 and 9p21/CDKN2A, alongside characteristic modifications in FOXP1 and BCL2. These genomic features are largely shared with diffuse large B-cell lymphoma (DLBCL) in the general population (2, 56). Furthermore, critical molecular alterations such as TP53 mutations—which are frequent in EBV-negative disease but notably absent in early lesions—along with MYC rearrangements and NOTCH1 mutations, confer an autonomous and aggressive proliferative capacity independent of viral oncogenic control (55). At the protein level, EBV-negative cases more frequently exhibit a germinal center B-cell (GCB) immunophenotype and are typically CD30-negative and p53-positive by immunohistochemistry (57, 58).

The TME of EBV-negative PTLD also differs significantly, showing lower regulatory T-cell (Treg) infiltration and lacking the robust expression of the type I interferon pathway and antiviral-response genes that characterize EBV-driven disease (55). In EBV-negative cases, NK cells tend to overexpress Tim-3 rather than showing the profound lymphopenia and PD-1 expression observed in the EBV-positive subtype. In this clinical context, CD4 lymphopenia (CD4 < 300 cells/mm³) has emerged as a significant prognostic factor, predicting poor 2-year progression-free survival (30). Ultimately, the EBV-negative TME is shaped not by viral cytokines like viral IL-10, but rather by persistent antigenic stimulation from the graft and cumulative immunosuppressive pressure (59).

### 3.3 Organ-specific variations in the PTLD tumor microenvironment

The TME in PTLD exhibits significant variations among different solid organ transplant (SOT) recipients, reflecting distinct immunological contexts, disease onset timing, and organ-specific immunosuppressive pressures. The median time to PTLD development varies markedly depending on the transplanted organ (60). This temporal disparity suggests divergent pathogenic mechanisms: early-onset PTLD (which occurs more frequently in lung and liver recipients) is predominantly EBV-driven and characterized by a more robustly inflammatory TME, whereas late-onset PTLD (a typical clinical course for kidney transplant recipients) likely reflects the consequences of cumulative pharmacological immunosuppression and acquired genomic instability (60).

While comprehensive organ-by-organ comparisons of the PTLD microenvironment within the SOT cohort remain limited, current evidence highlights that PTLD following SOT differs substantially from cases occurring after hematopoietic stem cell transplantation (HSCT) regarding immune cell infiltration and macrophage polarization (25). Within SOT recipients, the specific cellular composition of the TME is heavily influenced by the transplanted organ itself, as well as organ-specific immunosuppressive regimens, the intensity and duration of therapy required to prevent rejection, and the primary disease that necessitated the transplant (60). The type and intensity of immunosuppression differ significantly across organ types and directly modulate the TME. For instance, kidney transplant recipients generally receive lower levels of maintenance immunosuppression compared to lung, heart, or liver recipients. This lower intensity may contribute to the characteristically delayed onset of disease and potentially lead to a distinct immune infiltrate composition in PTLD associated with kidney transplantation (60, 61).

## 4 Treatment strategies and new therapeutic frontiers

### 4.1 Standard treatment strategies

As previously described, the pathogenesis of PTLD is extremely complex and heterogeneous, involving both viral-driven mechanisms and intricate alterations within the TME. Consequently, numerous emerging therapies aim not only to halt disease progression but also to reverse the immunosuppressive modifications in the TME discussed above. These modern targeted therapies are generally employed if the initial Reduction of Immunosuppression (RIS) fails to control the disease (62).

To date, RIS remains the cornerstone of first-line management. This strategy aims to restore the recipient’s immune surveillance against the lymphoproliferative cell pool and proves particularly effective in early-onset PTLD cases, which are often EBV-driven and retain some degree of immunogenicity. The standard RIS protocol typically involves a 30%–50% dose reduction of CNIs, such as tacrolimus or cyclosporine, alongside the discontinuation of antimetabolite agents like azathioprine or mycophenolate mofetil (62).

An alternative or complementary strategy involves reducing or substituting CNIs with inhibitors of the mTOR, such as sirolimus or everolimus. These agents offer a dual benefit: they lower the overall immunosuppressive burden compared to CNIs and exert direct anti-tumor effects. Their protective role is attributable to potent antiproliferative and immunomodulatory properties, which limit the expansion of EBV-positive B cells and dampen chronic immune activation, potentially disrupting the supportive signals within the TME (63).

In conjunction with these strategies, Rituximab constitutes the established first-line treatment for CD20-positive PTLD that remains unresponsive to initial immunosuppression reduction alone. This monoclonal antibody, directed against the CD20 surface antigen, has significantly improved outcomes in B-cell PTLD. Furthermore, for high-risk patients or those who do not achieve complete remission with Rituximab monotherapy, the treatment protocol is typically escalated to include a standard chemotherapy regimen, most notably CHOP (Cyclophosphamide, Doxorubicin, Vincristine, and Prednisone), often administered in a risk-stratified sequential manner (64, 65).

### 4.2 Adoptive cellular therapies and novel targeted agents

Adoptive cellular therapies represent an area of intense research aimed at specifically restoring immune control, which is deficient in immunosuppressed patients. EBV-Specific Cytotoxic T Lymphocytes (EBV-CTLs) are recognized as a promising approach to reconstitute immune surveillance, particularly in rituximab-refractory or multiply relapsed disease. These cells are obtained by stimulation with EBV-transformed B lymphoblastoid cell lines or viral vectors and selected via TCR tetramer binding or interferon-gamma capture. While initial studies utilized autologous CTLs, which are effective but require weeks to generate, recent strategies have focused on third-party allogeneic CTLs derived from healthy, HLA-matched, EBV-seropositive donors. These banks allow for rapid access to therapy (66).

Tabelecleucel (Tab-cel), the first regulatory-approved EBV-targeting cellular therapy, is an advanced, allogeneic EBV-specific T-cell immunotherapy targeting the Type III EBV latency profile, which is commonly expressed in PTLD. This makes it particularly suitable for early/polymorphic PTLDs that express this profile. By specifically recognizing EBV-infected B cells, these CTLs induce cytotoxicity via the perforin-granzyme pathway, sparing normal tissues. In the multicenter phase 3 ALLELE study, Tabelecleucel demonstrated a 51.2% overall response rate (ORR) in patients with relapsed/refractory PTLD, with a 1-year overall survival of 56% in solid organ transplant recipients. Crucially, this therapy showed an excellent safety profile with no evidence of treatment-related mortality or allograft rejection (67, 68).

CD19-directed chimeric Antigen Receptor (CAR) T-Cell Therapy has emerged as a promising option for relapsed/refractory PTLD, particularly the monomorphic diffuse large B-cell lymphoma (DLBCL) subtype, which frequently expresses CD19. In a systematic review of solid organ transplant recipients treated with CAR-T, the overall response rate was 82%, with a complete response rate of roughly 59%. However, the therapy presents significant challenges, including Cytokine Release Syndrome (CRS) and neurotoxicity. Furthermore, the interruption of immunosuppression often required for CAR-T efficacy poses a risk of allograft rejection, which occurred in approximately 23% of cases in one review (69, 70). Larger prospective studies are necessary to optimize safety protocols and determine the ideal timing of immunosuppression discontinuation.

### 4.3 Checkpoint inhibition

In certain tumors, particularly EBV-positive PTLD, PD-L1 (often driven by the viral oncoprotein LMP1) is frequently upregulate to evade immune recognition, making the PD-1/PD-L1 axis a rational therapeutic target (71). However, the clinical application of Immune Checkpoint Inhibitors (ICIs) (e.g., Nivolumab, Pembrolizumab) in transplant recipients is severely limited by safety concerns. The blockade of PD-1 or CTLA-4 carries a high and documented risk of triggering allograft rejection (reported in up to 40% of cases) and graft failure (72). Consequently, their use is currently restricted to strictly experimental settings or patients without a functioning allograft.

### 4.4 Targeting the B-cell receptor pathway

Disrupting the B-cell receptor signaling pathway has emerged as a key strategy, particularly through the use of Bruton tyrosine kinase (BTK) inhibitors like ibrutinib (73–75). Preclinical models indicate that BCR signaling is indispensable for the expansion of EBV-positive plasmablasts that drive PTLD-like pathologies (74). Clinically, the phase 2 TIDaL trial investigated ibrutinib as a first-line agent alongside risk-stratified therapy. While the interim complete response rate was relatively low at 29%, the study demonstrated a 2-year overall survival of 76%, suggesting ibrutinib may provide durable disease control in selected cohorts (73). Furthermore, case reports have highlighted the successful use of ibrutinib in refractory EBV-associated primary CNS PTLD, especially when integrated with EBV-specific T-cell therapies (75).

### 4.5 Epigenetic modulators

Epigenetic therapies, including histone deacetylase (HDAC) inhibitors (e.g., romidepsin, belinostat) and DNA methyltransferase (DNMT) inhibitors (e.g., azacitidine), are gaining traction, particularly for T-cell PTLD where traditional chemotherapy often fails (2, 76, 77). The combination of romidepsin and azacitidine has shown high efficacy in peripheral T-cell lymphomas, with overall response rates between 73% and 76% (77). This synergy is driven by profound transcriptional remodeling, including the upregulation of cancer testis antigens and immune-modulatory pathways, providing a strong biological rationale for its application in the transplant setting (78).

### 4.6 BCL-2 inhibition and pro-apoptotic strategies

The selective BCL-2 inhibitor venetoclax represents another promising avenue, especially for cases with high BCL-2 expression (79, 80). Preclinical research has demonstrated that venetoclax synergizes with the proteasome inhibitor bortezomib to target the pro-survival functions of EBV latent proteins, specifically LMP-1 and EBNA-3C. By simultaneously inhibiting BCL-2 and MCL-1 while suppressing NF-κB signaling, this combination effectively induces apoptosis in EBV-transformed cells, suggesting a potential role in treating relapsed or refractory EBV-driven PTLD (81).

## 5 Discussion and conclusion

PTLD represents a paradigmatic example of how oncogenic viruses exploit iatrogenic immunodeficiency, yet current evidence suggests a far more complex pathogenesis than previously understood, highlighting a fundamental conceptual shift where PTLD is no longer viewed merely as a passive consequence of systemic T-cell depletion but as a dynamic malignancy driven by the ability of EBV-infected B cells to actively engineer a sophisticated, immune-privileged TME (11, 12). We propose here a hierarchical model of immune evasion where EBV-infected B cells actively engineer their niche through three distinct functional levels. At the foundation of this hierarchy lies the recruitment phase: orchestrated by Latency III programs and LMP1, the tumor initiates the production of viral cytokines (vIL-10) and chemokines (CCL3/4/5) to recruit MDSCs and Tregs (11, 14, 21). The second level involves metabolic remodeling: once recruited, these suppressive cells create a biochemical barrier through L-arginine depletion and ROS production, preventing T-cell activation (15, 17, 19). Finally, the terminal level is the ‘molecular execution’ of immunosuppression, where PD-L1 expression and exosomal miRNAs act as the final brakes on effector cells, contributing to the pronounced biological heterogeneity observed between subtypes (25, 38, 62–64). This heterogeneity, coupled with the “fundamental clinical dilemma” of balancing potent anti-tumor efficacy with the preservation of the transplanted organ, remains a central challenge in modern management; indeed, while reduction of immunosuppression and novel immune checkpoint inhibitors are biologically rational, they inherently increase the risk of acute or chronic graft rejection (2, 9, 56, 60). Consequently, future research must pivot toward precision medicine approaches that utilize refined sub-classification and precision diagnosis (incorporating molecular profiling, liquid biopsies for circulating tumor DNA, and spatial transcriptomics) to stratify patients based on their specific genomic drivers and mechanisms of immune evasion (61–64). In this context, future therapeutic directions should emphasize combination strategies that integrate targeted small-molecule agents, such as BTK or BCL-2 inhibitors, with graft-sparing conventional or cellular therapies, alongside the development of preventive approaches based on high-sensitivity EBV-DNA monitoring and the identification of early pre-malignant TME signatures (1, 13, 43, 65). Ultimately, dismantling the tumor’s protective niche through a personalized framework that accounts for organ-specific pressures and viral status will be critical to ensuring that the pursuit of oncological cure does not jeopardize the long-term survival of the life-saving graft.

## Glossary

ATG: anti-thymocyte globulin
BTK: Bruton tyrosine kinase
CAR: chimeric antigen receptor
CCL: chemokine (C-C motif) ligand
CCR5: C-C chemokine receptor type 5
CHOP: cyclophosphamide, doxorubicin, vincristine, and prednisone
CNI: calcineurin inhibitors
CRS: cytokine release syndrome
CTAR: C-terminal activation region
ctDNA: circulating tumor DNA
DLBCL: diffuse large B-cell lymphoma
DNMT: DNA methyltransferase
EBERs: Epstein-Barr virus-encoded RNAs
EBNA: Epstein-Barr nuclear antigen
EBV: Epstein–Barr virus
EBV-CTLs: EBV-specific cytotoxic T lymphocytes
EVs: extracellular vesicles
GCB: germinal center B-cell
HDAC: histone deacetylase
HLA: human leukocyte antigen
HSCT: hematopoietic stem cell transplantation
ICIs: immune checkpoint inhibitors
IDO: indoleamine 2,3-dioxygenase
IL: interleukin
iNOS: inducible nitric oxide synthase
KT: kidney transplantation
LMP: latent membrane protein
M-MDSCs: monocytic myeloid-derived suppressor cells
MDSCs: myeloid-derived suppressor cells
miRNAs: microRNAs
MMF: mycophenolate mofetil
mPTLD: monomorphic PTLD
mTOR: mammalian Target of Rapamycin
NF-κB: Nuclear Factor kappa-light-chain-enhancer of activated B cells
NK: natural killer
NO: nitric oxide
ORR: overall response rate
PD-1: programmed death protein 1
PD-L1: programmed death-ligand 1
PTLD: post-transplant lymphoproliferative disorders
RIS: reduction of immunosuppression
ROS: reactive oxygen species
SOT: solid organ transplant
TAMs: tumor-associated macrophages
TCR: T-cell receptor
TGF-β: transforming growth factor-β
TME: tumor microenvironment
Treg: regulatory T-cell
WHO: World Health Organization
